# Spectral tomographic imaging with aplanatic metalens

**DOI:** 10.1038/s41377-019-0208-0

**Published:** 2019-11-06

**Authors:** Chen Chen, Wange Song, Jia-Wern Chen, Jung-Hsi Wang, Yu Han Chen, Beibei Xu, Mu-Ku Chen, Hanmeng Li, Bin Fang, Ji Chen, Hsin Yu Kuo, Shuming Wang, Din Ping Tsai, Shining Zhu, Tao Li

**Affiliations:** 1 National Laboratory of Solid State Microstructures, Key Laboratory of Intelligent Optical Sensing and Integration, Jiangsu Key Laboratory of Artificial Functional Materials, College of Engineering and Applied Sciences, Nanjing University, Nanjing, 210093 China; 2 Collaborative Innovation Center of Advanced Microstructures, Nanjing, China; 3 Research Center for Applied Sciences, Taipei, 11529 Taiwan, China; 4 Department of Physics, Taiwan University, Taipei, 10617 Taiwan, China; 5 Graduate Institute of Electronics Engineering, Taiwan University, Taipei, 10617 Taiwan, China

**Keywords:** Imaging and sensing, Metamaterials

## Abstract

Tomography is an informative imaging modality that is usually implemented by mechanical scanning, owing to the limited depth-of-field (DOF) in conventional systems. However, recent imaging systems are working towards more compact and stable architectures; therefore, developing nonmotion tomography is highly desirable. Here, we propose a metalens-based spectral imaging system with an aplanatic GaN metalens (NA = 0.78), in which large chromatic dispersion is used to access spectral focus tuning and optical zooming in the visible spectrum. After the function of wavelength-switched tomography was confirmed on cascaded samples, this aplanatic metalens is utilized to image microscopic frog egg cells and shows excellent tomographic images with distinct DOF features of the cell membrane and nucleus. Our approach makes good use of the large diffractive dispersion of the metalens and develops a new imaging technique that advances recent informative optical devices.

## Bioimaging: metalenses make 3D analysis effortless

An ultrathin “metalens” that uses semiconductor nanostructures to focus light can help microscopes resolve detailed images of live cells without complex mechanical components. In conventional tomographic imaging, a scanning instrument passes over the sample and takes multiple cross-sectional images, which are then reconstructed into a 3D picture. Tao Li from Nanjing University in China and colleagues have now simplified this technique by fabricating lenses patterned with concentric rings of gallium nitride nanopillars. The lens made of nanopillars has wavelength-dependent properties that enable focusing on different regions of a target simply by sweeping through the visible light spectrum. Experiments with frog egg cells demonstrated this approach could zoom in and obtain detailed, high quality images of the inner membrane and nucleus without harming the sample or moving the metalens.

## Introduction

Recent advances in flat optics, enabled by metasurfaces with flexible control of the amplitude, phase, and polarization of light by subwavelength units^1–6^, have provided unprecedented possibilities in the miniaturization and function expansion of conventional optical elements. In addition to many potential applications of metasurfaces, such as holograms^7,8^, wave plates^9,10^ and invisible cloaking carpets^11,12^, the metalens has captured significant attention in recent years due to its extremely wide usage in optical systems^13–29^. Great efforts have been made towards practical applications, such as efficiency improvement^20^, chromatic aberration correction^21–26^ and image corrections^27,28^. Although these progresses definitely bring the metadesign closer to the application level, tunable metasurface devices remain a great challenge^29,30^. In fact, inheriting the diffraction dispersion, a metalens usually has large chromaticity, which possibly allows us to utilize the wavelength as a tuning dimension to access tunable functionalities. Specifically, the large chromatic dispersion of a metalens makes the focal length tunable with respect to the wavelength, and one may access a nonmotion optical zooming and depth-of-field (DOF) spanning by simply switching the illumination wavelength. Therefore, spectral tomography would be implemented by wavelength sweeping to image a certain DOF-spanned object in three dimensions.

Conventional tomography is usually implemented by mechanical scanning (e.g., confocal microscopy^31,32^) with complicated mechanical components. A nonmotion design for tomography with more efficient and stable performance would be quite desirable. Because of their varied focal lengths, chromatic optical lenses have been utilized to extend the DOF of imaging^33^, which makes it promising for nonmotion spectral tomography. Several works have been reported in chromatic confocal imaging or surface profiling^34–40^. Compared with a refractive lens, a diffractive lens has much larger chromatic dispersion^41^, which is beneficial for broader DOF spanning. Moreover, planar diffractive devices reduce the amount of space needed and thus favor compact integration. However, these conventional diffractive optical elements^37,38^ (i.e., Fresnel lens), as used for imaging purposes, usually suffer from several shortcomings, such as low efficiency^42^, the shadowing effect^43^, and low signal-to-noise ratio (SNR), which greatly prohibits their imaging applications.

In this paper, we report the design and implementation of an aplanatic metalens with chromatic dispersion to achieve high-resolution spectral tomographic imaging in a nonmotion manner. A high-numerical-aperture (NA = 0.78) metalens is experimentally demonstrated to obtain a high imaging resolution (775 nm) within the visible wavelength range (450–660 nm). The aplanatic design for a certain imaging distance guarantees a high longitudinal resolution and is confirmed by imaging a group of rotated hole slides cascaded along the optical axis. This aplanatic metalens is used for the microscopic tomography of frog egg cells, which shows a clear evolution of the defocus–focus–defocus process and fine resolution such that one can distinguish the depths of the cell membrane and nucleus. The priority of the metalens compared with the conventional diffraction Fresnel lens is also discussed. Our approach will quite possibly open up a new door to intriguing applications of the metalens that were previously limited by conventional optical designs.

## Results

### Aplanatic phase design

High transverse resolution and high longitudinal resolution are both indispensable for tomographic imaging. As we know, high NA is exceedingly beneficial to high resolution in both the transverse direction (0.61*λ/*NA by Rayleigh criterion^44^) and the longitudinal direction (${\mathrm{DOF}} = {\mathrm{\lambda /}}(1 - \sqrt {1 - {\rm{NA}}^2} )$^45^). For a large-NA lens, spherical aberration also plays an important role in both the transverse and longitudinal resolutions. In common cases, the phase profile of a focal metalens is defined by

1 $$\varphi = \frac{{2\pi }}{\lambda }(f - \sqrt {R^2 + f^2} )$$

where *R* is the position in the radius dimension and *f* is the focal length. It is derived from the plane wave incidence, and there is no spherical aberration for the flat metalens^46^. However, in cases of microscopic imaging, the object is quite close to the lens, and the objective field tends to be a spherical wave instead of a plane wave. Therefore, using a normal metalens will cause large spherical aberrations in microscopic imaging, which definitely degrades the image quality. To correct the spherical aberration with respect to the high-NA metalens, we introduced an aplanatic phase profile as a function of the working distances based on the generalized laws of refraction and imaging^1^ as

2 $$\varphi = \frac{{2\pi s}}{{\lambda \left( {f - s} \right)}}\left( { - \frac{{\left( {f - s} \right)\sqrt {s^2 + R^2} }}{s}\, + \,\frac{{\sqrt {\left[ {2f^2 - 2fs + s^2 + \left( {2f - s} \right)\left( {\frac{{2s^2}}{{s^2 + R^2}} - 1} \right)} \right]\frac{{s^2 + R^2}}{{s^2}}} }}{{\sqrt 2 }}} \right)$$

where *s* is the object distance and *λ* is the working wavelength. This is a generalized aplanatic phase profile; when *s* approaches infinity, it is simplified to Eq. (1). Here, to capture tomographic images as accurately as possible, we choose *s* = 2 *f*; then, the aplanatic phase profile becomes

3 $$\varphi = \frac{{4\pi }}{\lambda }\left( {2f - \sqrt {R^2 + 4f^2} } \right)$$

**Fig. 1:**
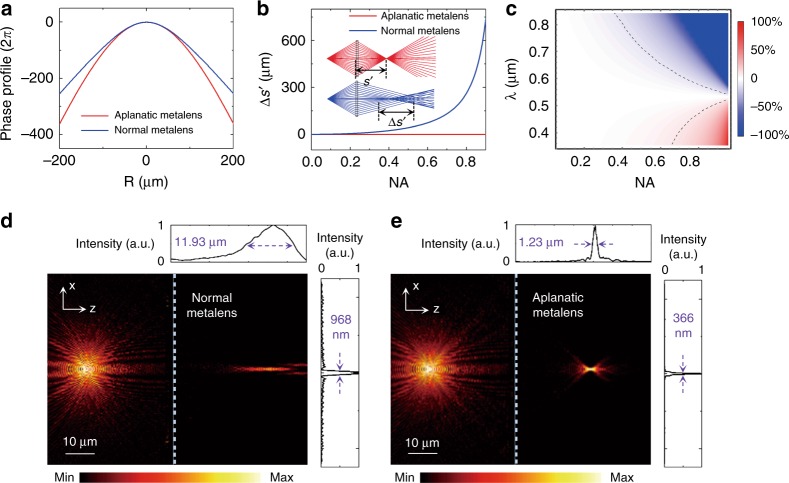
Aplanatic metalens design. **a** Phase profiles designed for a normal (blue) and an aplanatic (red) metalens. **b** Spherical aberration (Δs′) of the normal and aplanatic metalenses as a function of the numerical aperture (NA), where the inset figures are the corresponding ray tracing results with a lens NA = 0.78 as an example. **c** Normalized spherical aberration (Δ*s*′*/s*′) as a function of the NA and wavelength, showing the broadband performance. **d**,**e** Full-wave simulation results of the point-source imaging with the normal and aplanatic metalenses, respectively

The phase profiles of normal and aplanatic metalenses are shown in Fig. 1a, where the parameters are set as *f* = 80 μm at *λ* = 532 nm. Figure 1b shows the comparison of spherical aberration (Δ*s*′) between these two kinds of metalens when the object distance *s* = 2 *f* = 160 μm with respect to different NA. It is apparent that as the NA increases, the corresponding Δ*s*′ of the normal metalens (blue curve) increases dramatically, while that of the aplanatic metalens (red line) remains at zero. This phenomenon can be clearly manifested by the ray traces of light with a Zemax simulation, as shown in the inset of Fig. 1b (with NA = 0.78), in which all light rays from different incident angles converge at the same point for the aplanatic lens, while the normal lens shows obvious divergence. Although the aplanatic phase profile is designed for a fixed wavelength (532 nm), it still works well in a broadband spectrum. Figure 1c illustrates the theoretical results of normalized spherical aberration (Δ*s*′/*s*′) as a function of the wavelength *λ* and lens NA. The region inside the dotted lines indicates that the change in Δ*s*′/*s*′ is lower than 10%, corresponding to a good imaging performance.

To confirm the theoretical analyses, full-wave simulations were performed by a commercial solution (CST Microwave Studio). Here, *f* is reduced to 10 μm, and *s* is set as 20 μm in the simulations to avoid an unnecessary large computation time. Figure 1d shows the 2D axial point imaging with a normal metalens, where to the left is the point source and to the right is the point image, with its transverse and longitudinal full width at half maximum (FWHM) being 968 nm and 11.93 μm, respectively. However, those of the aplanatic metalens are 366 nm and 1.23 μm, respectively, as shown in Fig. 1e. It is evident that the aplanatic lens exhibits greatly improved focusing capability for a certain point source in the 2 *f* distance, which indicates that a high resolution can be achieved as it is utilized for tomographic imaging.

### Characterization of the aplanatic metalens

**Fig. 2:**
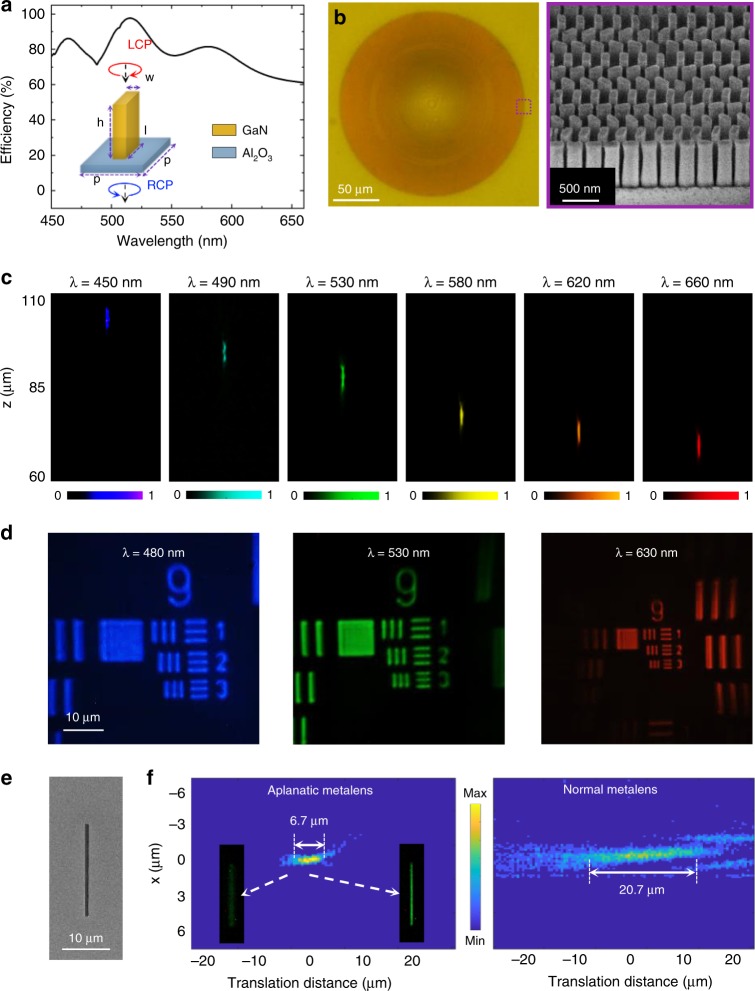
Experimental characterization of the aplanatic metalens. **a** Calculated conversion efficiency of the optical field by the unit cell in the working wavelength range. **b** Optical and SEM images of the fabricated metalens with NA = 0.78. **c** Experimental longitudinal cross section of the focusing light intensity by a metalens at different wavelengths in the plane wave incidence. **d** Images of the USAF resolution test chart with the metalens at different wavelengths. **e** SEM image of a nanoslit sample. **f** Longitudinal cross section of the moving slit as a 2D point source imaged with the aplanatic (left) and normal (right) metalenses

As a proof of concept, an aplanatic metalens based on Eq. (3) with NA = 0.78 at *λ* = 532 nm with the Pancharatnam–Berry (PB) phase design^47,48^ was fabricated with gallium nitride (GaN) nanopillars on a sapphire substrate^25^ (see Methods). GaN was selected here because it is a low-loss semiconductor material over the entire visible spectrum (the band gap is approximately 3.4 eV, equal to the wavelength of 364.67 nm^49^). The period of the unit cell is chosen as *p* = 240 nm, which is smaller than the equivalent wavelength range (450–660 nm) in the substrate (450 nm/*n*_Al2O3_ = 253 nm) but greater than the diffraction condition (660 nm/2*n*_Al2O3_ = 187 nm) to suppress higher-ordered diffractions. A nanopillar with a height of 800 nm is designed and fabricated after careful optimizations, and the length and width of the nanopillar are 200 nm and 100 nm, respectively, to maintain the anisotropy allowed by fabrication. Figure 2a depicts the calculated conversion efficiency within the band of 450–660 nm with an average efficiency of 79%. Figure 2b shows the optical image and scanning electron microscope (SEM) images of this metalens with a diameter of 200 μm.

First, the chromatic dispersion of the aplanatic metalens was demonstrated as it was illuminated by a white-light laser (Fianium Super-continuum, 4 W) with a wavelength ranging from 450 to 660 nm. A linear polarizer (LP) and quarter-wave plate (QWP) were employed to generate the circular polarization incidence, and another LP and QWP, for cross-polarization analyses after the metalens. To capture the light intensity profile, an achromatic objective (×100, NA = 0.70) and a coupled charge device (CCD) were placed on a motorized stage and moved together along the propagation direction. The measured cross-sectional intensities of the focusing light are partially displayed in Fig. 2c. It is observed that the focal length changes from 102.7 to 69.2 μm as the incident wavelength sweeps from 450 to 660 nm, showing a large focal length change of 42% compared with that of the center wavelength (*λ* = 532 nm). Due to the limited NA of the collecting objective (×100, NA = 0.70), we did not precisely measure the efficiency in the experiments.

Next, the 1951 United States Air Force (USAF) resolution test chart was employed to test the lens resolution. Instead of the super-continuous laser, a halogen light (incoherent) source is used for the illumination, with filters of 10 nm bandwidth to acquire clear images and avoid speckle noises. The sample was directly mounted approximately *s* = 2 *f* = 160 μm in front of the metalens, corresponding to a 4 *f* optical configuration without the image zoom (here, *f* = 80 μm at *λ* = 530 nm). Figure 2d shows the microscopic images of the USAF resolution test chart with filters of *λ* = 480, 530, and 630 nm from left to right, manifesting clear resolution of Element 3 and Group 9 (i.e., a resolution of 775 nm). As illustrated in Fig. 1c, this aplanatic design has a considerable bandwidth that guarantees this high transverse resolution (≤775 nm) in the whole concerned wavelength range (450–660 nm) (more detailed data are provided in the Supporting Information, section S1), indicating the capability of working in the broadband for the spectral tomography function. As expected, this high resolution obtained from the aplanatic lens design also shows its advantage compared with the normal metalens, as illustrated in the Supporting Information, section S2.

The longitudinal resolution is another important index in our spectral imaging process. Thus, a single nanoslit of 800 nm in width fabricated by a focus ion beam (FIB, dual-beam FEI Helios 600i) was used as a 2D point source to measure the DOF (here, the image distance is fixed at *s*′ = 2 *f* = 160 μm). The SEM image of the slit is shown in Fig. 2e. This slit sample was directly placed in front of the metalens and mounted onto an electric translation stage to carefully adjust its position, and then a series of images obtained using the metalens were captured with different object distances. Figure 2f (left) displays the longitudinal map of the middle of the slit imaged by the aplanatic metalens with respect to the translation object distance (Δ*s*), which shows a relatively small DOF of approximately 6.7 μm (532 nm filter with a bandwidth of 3 nm). The clear and blurred inset figures correspond to the focused and defocused cases, respectively. However, for a normal metalens without an aplanatic design, a much larger DOF of 20.7 μm was observed, as shown in Fig. 2f (right). It qualitatively agrees well with our theoretical prediction in Fig. 1d, e (more data at other wavelengths can be found in the Supporting Information, section S1 and S2). This considerably high longitudinal resolution significantly enables this aplanatic metalens to work in a microscopic tomography.

### Spectral 3D tomographic Imaging

**Fig. 3:**
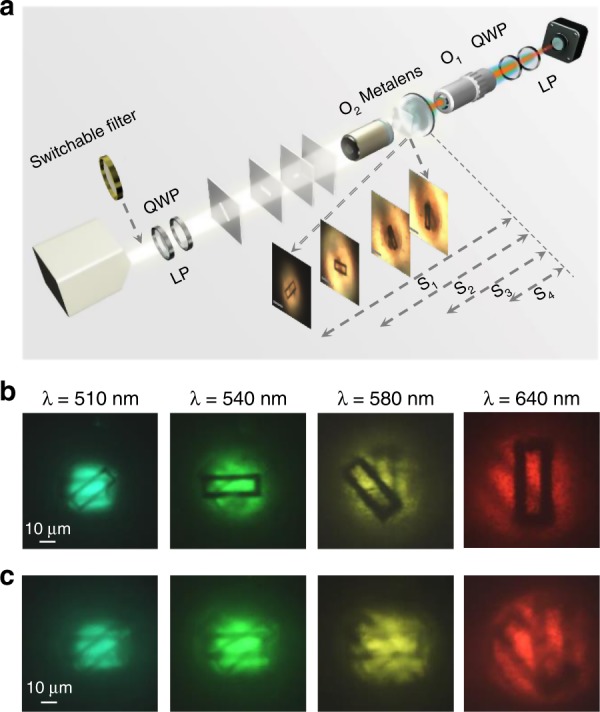
Spectral 3D tomographic imaging. **a** Schematics of the imaging setup. Lighting source is a halogen lamp. The four pictures in the inset are images through an achromatic objective O_2_ (×40, NA = 0.75) and are used as objects with different depths for the metalens to verify the tomographic imaging. The scale bar is 10 μm. The marked distances are measured distances from the metalens. The experiment captured images through an achromatic objective O_1_ (×100, NA = 0.70) and a CCD for the cases of (**b**) the aplanatic metalens and (**c**) the normal metalens for the same numerical aperture (NA = 0.78)

This aplanatic metalens based on the geometrical PB phase has demonstrated a large dispersed object distance with respect to wavelengths in the above resolution analyses; therefore, it is capable of resolving different DOF information of a 3D structured object. To clearly demonstrate this process, we prepared four slides with printed rectangular holes of different sizes and orientations, which are particularly arranged along the optical axis. Here, an additional object (O_2_) (×40, NA = 0.75) is used to translate the macroscopic slide objects to a series of microscopic second-order objects for the metalens imaging with the optical setup shown in Fig. 3a, where the translated object distances are indicated with respect to each slide microscopic image through O_2_. With the obtained knowledge of the focal length at each wavelength, it is time to obtain image information at different object depths without any moving elements. Figure 3b shows the images of the recorded rotated objects through the aplanatic metalens with corresponding wavelengths of 510 nm, 540 nm, 580 nm, and 640 nm from left to right, and the calculated object distances are 267.0 μm, 224.7 μm, 185.5 μm, and 147.0 μm, respectively, in which the errors are less than 4.5% (see Supporting Information Table S1). From these images, it is easy to distinguish each layer of the orientated holes, confirming the function of tomography. For comparison, we also measured a normal metalens without the aplanatic design for tomographic imaging. The corresponding results are shown in Fig. 3c (detailed comparison results within the wavelength range of 510–640 nm are provided in Supporting Information Fig. S7). It is quite evident that the images from different layers overlap, which indicates loss of the function of tomography. Obviously, the aplanatic design not only endows the metalens with much better imaging quality (i.e., higher resolution) but also shows its necessity in tomographic imaging.

### Microscopic spectral tomography of biocells

**Fig. 4:**
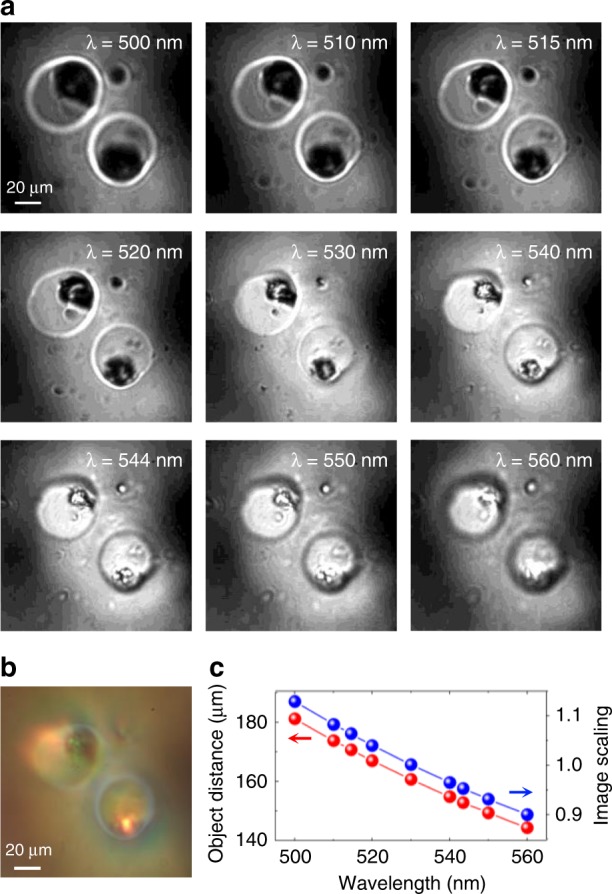
Microscopic spectral tomography of frog egg cells. **a** Microscopic images with the aplanatic metalens at different wavelengths. **b** Direct white-light image with the aplanatic metalens. **c** The derived layer positions and imaging scalings at different wavelengths

With the ability of the DOF resolution being confirmed, this dispersive aplanatic metalens is highly expected to image microscopic biological specimens via tomography. Here, we placed a specimen of frog egg cells directly in front of the metalens with a certain object distance. Figure 4a shows a group of microscopic images obtained by the metalens with different wavelengths from 500 to 560 nm, the colors of which have been removed; the sizes are normalized according to the zoom scaling to show the relatively realistic morphology and inner structures of the frog cells. The direct white-light image obtained by the metalens is given in Fig. 4b, showing a colorful picture due to the large dispersion. It is clear that the images of the cell membrane and nucleus evolve from blurry to clear and back to blurry again as the wavelength increases. Significantly, it is found that the cell nucleus changes from a large dark appearance to a small bright one, the change contrast of which is much stronger than that of the membrane images. This indeed indicates that the cell membrane and nucleus have different depths of field according to their different sizes. By a more careful comparison of these images, one may find that the clearest image of the cell membrane is at *λ* = 520 nm, while that is at *λ* = 530 nm for the nucleus, implying that there would be a small location distance between the layer centers of the membrane and nucleus. The derived imaging data of the layer position and imaging scaling with respect to the wavelengths are plotted in Fig. 4c, according to which the depth of the frog cell is roughly estimated to be 35 μm, while that of the nucleus is 5 μm. The detailed spectral images can be switched to another group by modulating the specimen position, which is compared with the results of Fig. 4a without dropping colors, as shown in the Supporting Information, section S3. From this information, we can also deduce the same morphologic information of the cell samples. Note that the frog egg cells are from a specimen that has already been flattened to some extent during preparation; thus, their morphologies are not kept spherical as was the case for the living ones.

## Discussions

Because of the large diffraction dispersion, use of the metasurface/metalens is always under debate owing to its large chromaticity when it works in the broadband range. Although there has been much important progress in the achromatic designs reported^21–26^, they still suffer from large scaling or limited use in small NA devices. In contrast, our approach utilizes the spectral dispersion as a new dimension to acquire more information in the imaging process, in which the large chromaticity of the metasurface is an advantage. On the other hand, conventional diffractive lenses also have large diffraction dispersions that can be designed for this kind of spectral tomography when adopting our aplanatic phase profile. To complete this research, we further fabricated normal and aplanatic diffractive binary Fresnel lenses and characterized their imaging performances carefully; see the detailed results and comparisons provided in the Supporting Information, section S4. Because of the multiple focuses, partially overwavelength structures, and polarization independence, the binary Fresnel lens manifests much worse imaging quality than that of the metalens (e.g., resolution, image sharpness and contrast, and SNR), although it has a similar spectral tomography function. In particular, according to our data, there is a ~7 dB decrease in the SNR for the binary Fresnel lens compared with the metalens (see Supporting Information Fig. S13). Additionally, we even compared several types of Fresnel lens and metalens efficiencies by theoretical simulation via the Lumerical-FDTD solver, the results of which are provided in Supporting Information Figs. S15 and S16. It is found that even the high-level (8-level) Fresnel lens still exhibits lower efficiency than that of the corresponding metalens. All of this supplementary information definitely shows the advantage of the metalens for this kind of tomographic imaging compared with conventional DOE devices.

It should be further mentioned that although the wavelength information has been extracted to achieve DOF-spanned imaging, it is still possible to restore colorful images by implementing the YUV color transformation on the captured RGB images^33^. This implies further upgrading of our tomography method, as it is used with assistance of advanced computational techniques. Moreover, our approach can also be adapted to those spectrum featured samples, and more complicated 3D spectral images with enriched information can be recorded one at a time to speed up the information collection and processing.

In conclusion, we have developed an aplanatic GaN metalens with a large numerical aperture (NA = 0.78) based on the PB phase design, the imaging properties of which were carefully characterized in a broad wavelength range in the visible spectrum. With the aplanatic design, high transverse and longitudinal resolutions were obtained, namely, approximately 775 nm and 6.7 μm, respectively. More interestingly, the large chromaticity that enables the function of spectral focus tuning, optical zooming, and tomographic imaging was revealed. As an example, microscopic tomographic imaging was carried out on frog egg cells, with clear manifestations of the morphologies and DOFs of the cell membranes and nucleus. Finally, the ultrathin and ultralight features of a metalens would be very helpful in developing ultracompact image devices and possibly lead to a breakthrough in terms of developing new conceptual imaging technologies.

## Materials and methods

### Sample preparation

An 800-nm-thick undoped GaN layer is grown on a double-polished sapphire by metalorganic chemical vapor deposition (MOCVD). A 400-nm-thick SiO_2_ layer is deposited through plasma-enhanced chemical vapor deposition (PECVD) as a hard mask layer to achieve a high aspect ratio. Next, the sample is spin-coated with a 100-nm-thick ZEP-520A e-beam resist layer, and then electron-beam lithography (EBL) with a 100-kV acceleration voltage is used to expose the sample to characterize the features of the structure. The pattern appears after the development process in ZEP-N50. Afterward, an electron beam evaporator (EBE) is used to deposit a 40-nm-thick Cr layer as a hard etching mask. We use a solution of *N*,*N*-dimethylacetamide (ZDMAC) to address the lift-off process. Then, we transfer the pattern to a SiO_2_ hard mask layer by reactive ion etching (RIE). Subsequently, Cr is removed by wet etching. After that, we continue to etch the patterned SiO_2_ hard mask layer by the inductively coupled plasma reactive ion etching (ICP-RIE) system. Finally, a GaN-based structure is obtained by removing the remaining SiO_2_ mask with a buffered oxide etch (BOE) solution^25^.

### Optical measurements

A broadband halogen lamp is employed as the illumination source. Filters with a bandwidth of 10 nm are used to characterize the features at each single wavelength. Quarter-wave plates (QWPs) and linear polarizers (LPs) are added to properly select the circular polarization state. Objective O_1_ (×100, NA = 0.70), the later QWP, the LP, and the CCD are introduced for imaging analyses. The object for imaging and the imaging acquisition system (O_1_ and CCD) are mounted onto two separated motorized stages, which can be carefully adjusted independently along the optical direction.

### Full-wave simulations

We use a CST Microwave Studio to simulate the 2D axial point imaging. A discrete port was used as the imaging source. Periodic boundary conditions were set in the *y* direction, and open boundary conditions were set in both the *x* and *y* directions.

## Supplementary information

Supplementary Information
