# Supplementary material for: Spectral tomographic imaging with aplanatic metalens
Source: Light Sci Appl. 2019 Nov 6;8:99. doi: 10.1038/s41377-019-0208-0 (PMC6834576; doi:10.1038/s41377-019-0208-0)
Supplement: Supplementary file 1 — Supplementary Information [file 41377_2019_208_MOESM1_ESM.docx]

**Supporting Information for**

**Spectral tomographic imaging with aplanatic metalens**

Chen Chen^1,2^, Wange Song^1,2^, Jia-Wern Chen^3,4^, Jung-Hsi Wang^5^, Yu Han Chen^3,4^, Beibei Xu^1,2^, Mu-Ku Chen^3,4^, Hanmeng Li^1,2^, Bin Fang^1,2^, Ji Chen^1,2^, Hsin Yu Kuo^3,4^, Shuming Wang^1,2^, Din Ping Tsai^3,4^, Shining Zhu^1,2^ and Tao Li^1,2^*

*^1^National Laboratory of Solid State Microstructures, Key Laboratory of Intelligent Optical Sensing and Integration, Jiangsu Key Laboratory of Artificial Functional Materials, College of Engineering and Applied Sciences, Nanjing University, Nanjing, 210093, China.*

*^2^Collaborative Innovation Center of Advanced Microstructures, Nanjing, China.*

*^3^Research Center for Applied Sciences, Taipei, 11529, Taiwan, China.*

*^4^Department of Physics, Taiwan University, Taipei, 10617, Taiwan, China.*

*^5^Graduate Institute of Electronics Engineering, Taiwan University, Taipei, 10617, Taiwan, China.*

**Corresponding author:* Prof. Tao Li, Email: [taoli@nju.edu.cn](mailto:taoli@nju.edu.cn) Tel: +86-25-83593805 URL: <http://dsl.nju.edu.cn/litao/>

**S1: Resolution test of the aplanatic metalens in broadband.**

The imaging resolutions with aplanatic metalens in a wide wavelength range (450 nm-660 nm) were characterized with filters of 10 nm bandwidth (see **Fig. S1**), revealing the resolution of 775 nm (element 3, group 9) within whole wavelength range.

We further characterized the depth-of-focus measurement by scanning the image distance with a fixed object distance. **Figure S2** shows the scanned longitudinal cross-section of the focused field with a filter in 532 nm (bandwidth of 3 nm), showing the depth of focus of 6.44 μm. For other wavelengths (filter with bandwidth of 10 nm), the results are provided in **Fig. S3.**  Noted that the depth of focus decreases with the increasing wavelength, which is due to the NA increases with wavelength. The small expansion around 530 nm accounts for the enlarged bandwidth of filter.

**
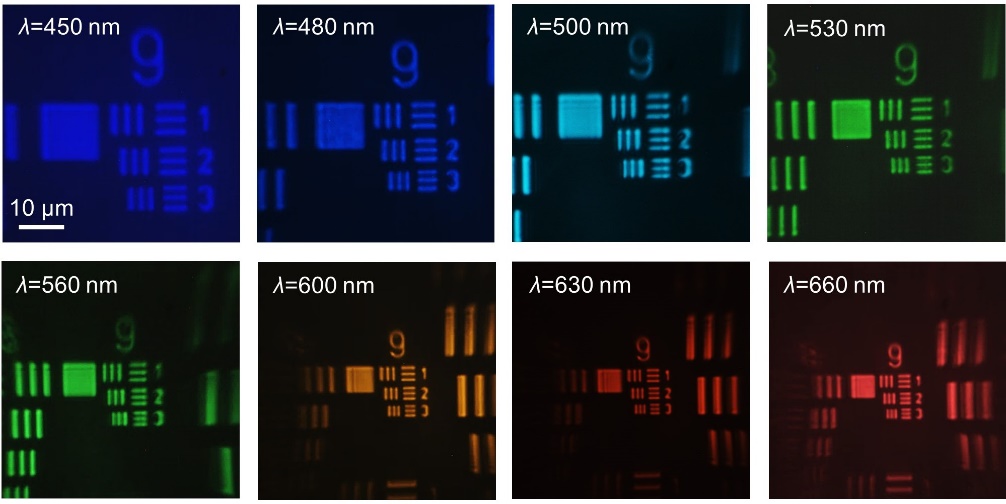
**

**Figure S1. Images of 1951 USAF resolution test chart with aplanatic metalens in the whole wavelength range (450 nm-660 nm).**

**
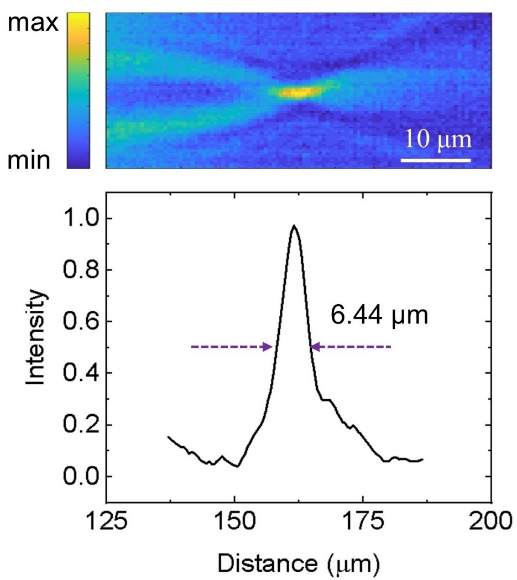
**

**Figure S2.** **Depth-of-focus characterization:** the longitudinal cross-section of focusing field with aplanatic metalens (532 nm filter, bandwidth of 3 nm).

**
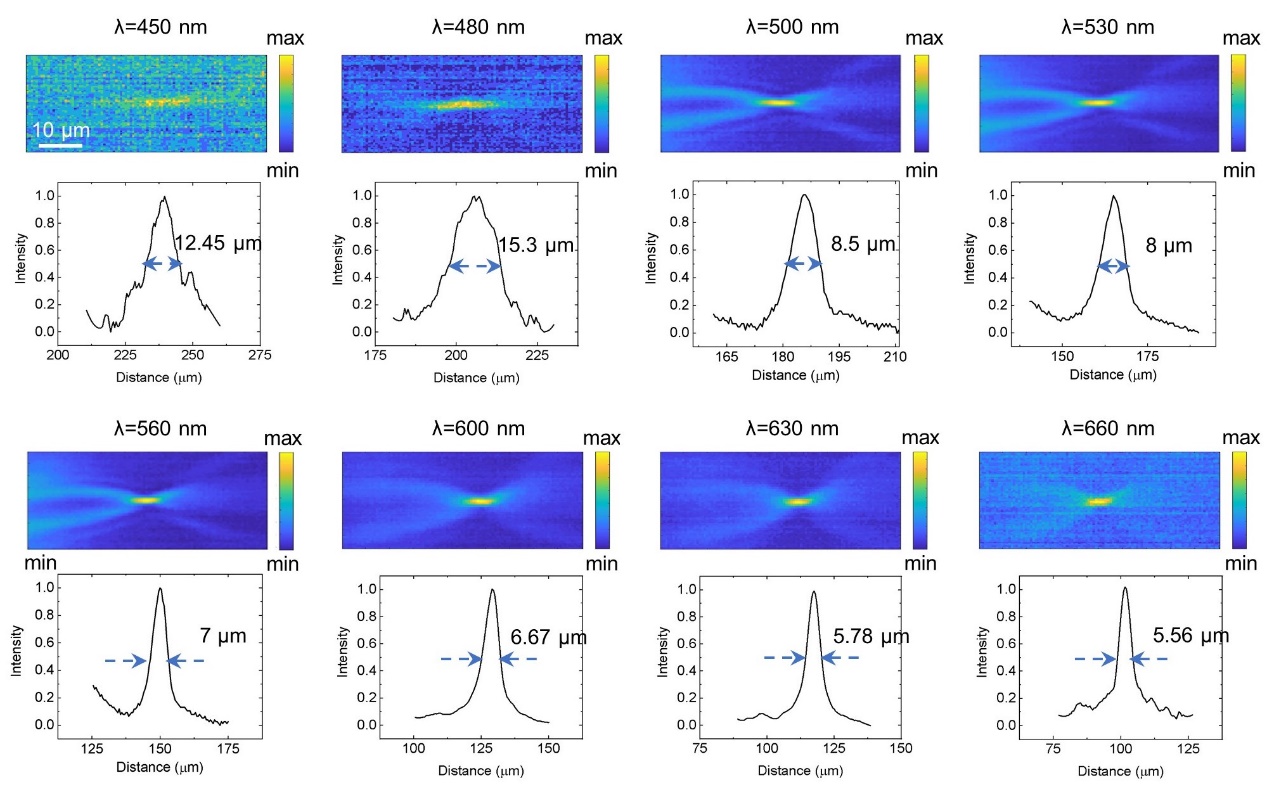
**

**Figure S3.** **Depth-of-focus characterization:** the longitudinal cross-section of focusing field with aplanatic metalens at different wavelengths (filter bandwidth of 10 nm).

**S2: Comparisons of imaging quality between the aplanatic metalens and the normal one.**

**Figure S4** shows imaged 1951 USAF resolution test chart with aplanatic and normal metalens respectively, where the aplanatic metalens shows the resolution of about 775 nm, while that of normal metalens is only 977 nm (Element 1, Group 9). The comparison of depth-of-field (DOF) and depth-of-focus results are provided in in **Figs. S5** and **S6**, both indicating better longitudinal resolution of the aplanatic metalens.

**
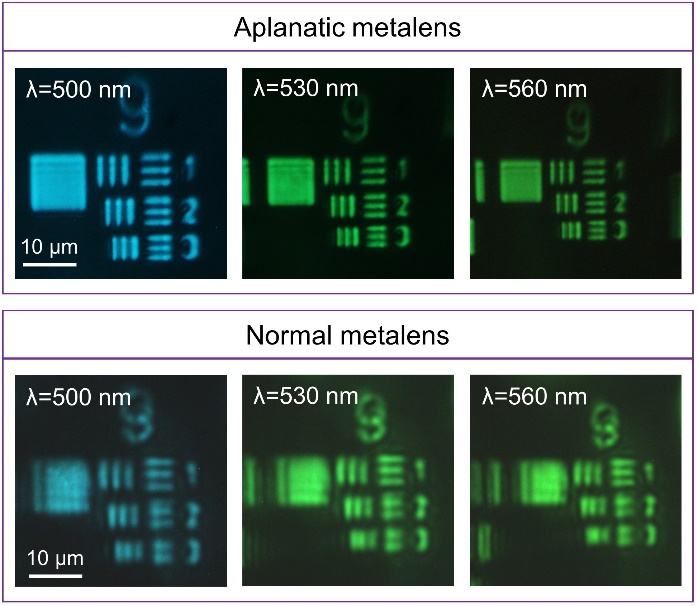
**

**Figure S4.** **Images of 1951 USAF resolution test chart with a,** the aplanatic metalens, and **b**, the normal metalens.

**
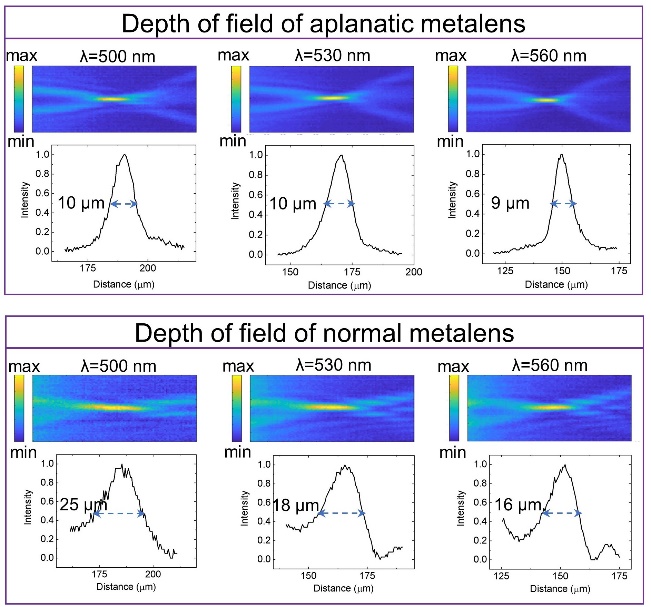
**

**Figure S5. Depth of field measurement results of aplanatic metalens and normal metalens for the longitudinal maps of the middle of the slit images.**

**
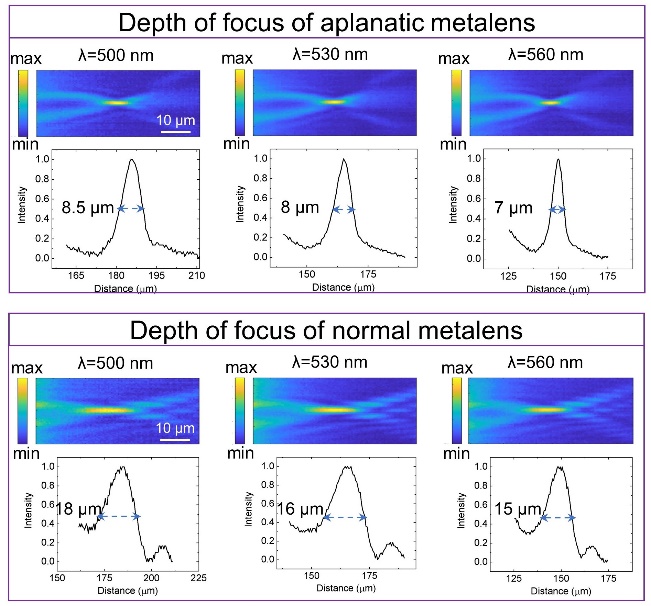
**

**Figure S6.** **Depth of focus measurement results of aplanatic metalens and normal metalens for the longitudinal maps of the middle of the slit images.**

**Figure S7** provides more detailed data for comparisons of tomographic imaging of four-layer samples, where the aplanatic metalens show less cross-talks between different imaging layers evidently. **Table 1** lists the data of detailed positions of imaging layers with respect to different wavelengths.


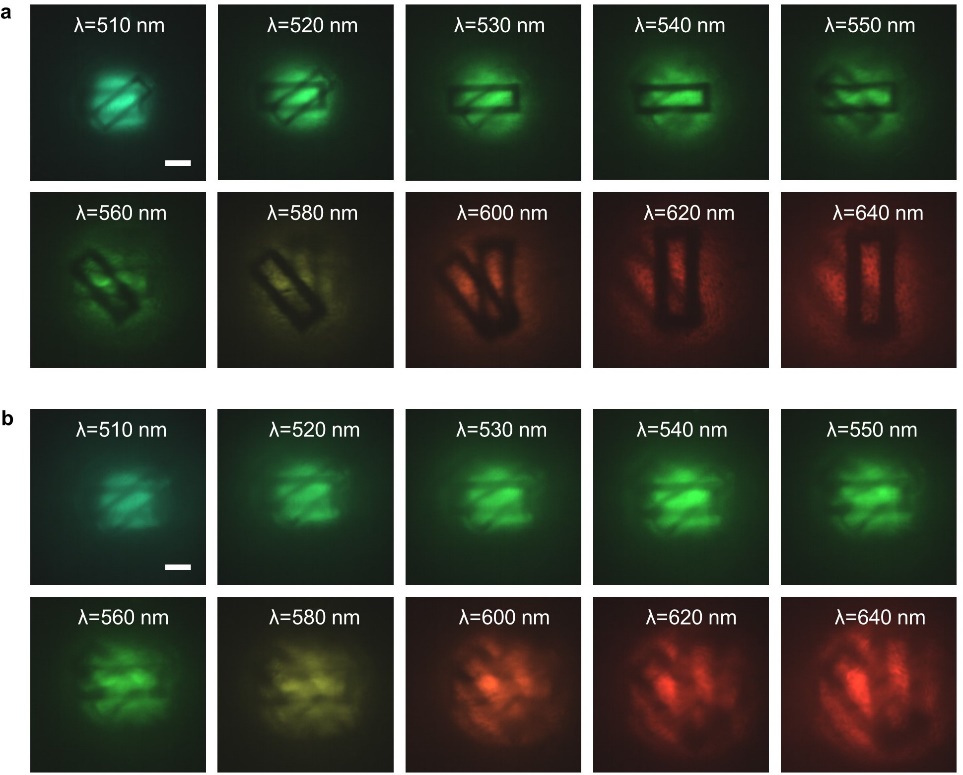


**Figure S7. Detailed experimental comparison between aplanatic metalens and normal metalens** **for the four-layer samples.** (The image distances are all the same as 121.4 μm.) Experimental results of **a,** aplanatic metalens, and **b,** normal metalens (scale bar is 10 μm).

**Table S1 | Experimental DOF results and analyses.**

| $\lambda(nm)$ | 510 | 540 | 580 | 640 |
| --- | --- | --- | --- | --- |
| Theoretical focal length (μm) | 83.45 | 78.81 | 73.38 | 66.50 |
| Retrieved object distance (μm) | 267.0 | 224.7 | 185.5 | 147.0 |
| Actual object distance (μm) | 270.5 | 235.3 | 180.2 | 150.1 |
| Error (%) | 1.29 | 4.50 | 2.94 | 2.07 |

**S3: Supplemental data of tomographic images of frog egg cells.**

**Figure S8** shows the microscopic images of frog egg cells at different layers by mechanical scanning with 2 μm per step, showing conventional tomographic images. **Figure S9** shows the detailed spectral tomography without dropping colors at different image distances. Both of them reveals the evolution from blurred cell membrane and nucleus to clear ones and return back to be blurred as the wavelength increases.


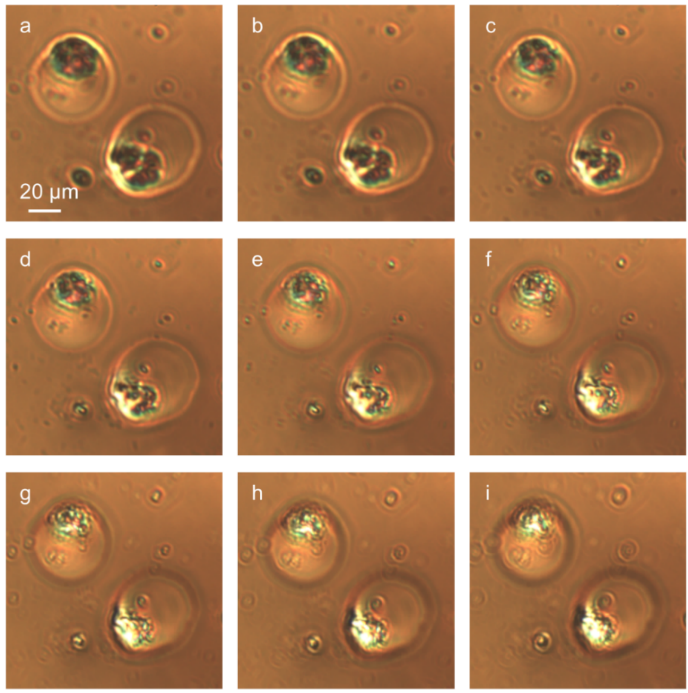


**Figure S8.** **Conventional microscopic images of the frog egg cells by mechanical moving with object distance moving 2 μm per step.**


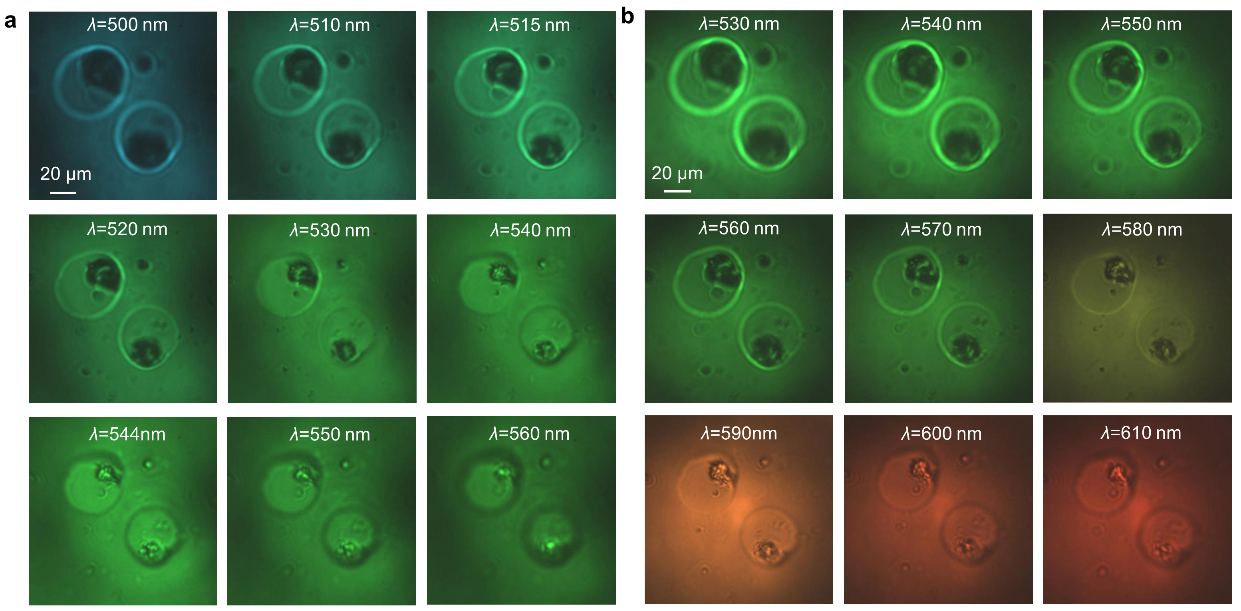


**Figure S9. More data of spectral tomography of frog egg cells.** Detailed spectral tomography with the aplanatic metalens with **a,** image distance of 160 μm, and **b,** image distance of 185.3 μm.

**S4: Comparisons between diffractive Fresnel lens and metalens**

For a reasonable and fair comparison, we fabricate two binary phase Fresnel lenses with normal and aplanatic designs respectively. The ring radii of normal Fresnel lens obeys

$r_{m}=\sqrt{m\lambda f+m^{2}\lambda^{2}/4}$, (S1)

while for the 4f-aplanatic lens, the ring radii follows

$r_{m}=\sqrt{\frac{{(4f+\frac{m\lambda}{2})}^{2}}{4}-4f^{2}}$. (S2)

The thicknesses of these Fresnel lenses all satisfy

$t=\frac{\lambda}{2(n_{SiO2}-1)}$ (S3)

Their diameters are both 200 μm with *NA*= 0.78 as same as the metalens. The SEM images of the aplanatic Fresnel lens are shown in **Fig. S10**.

**
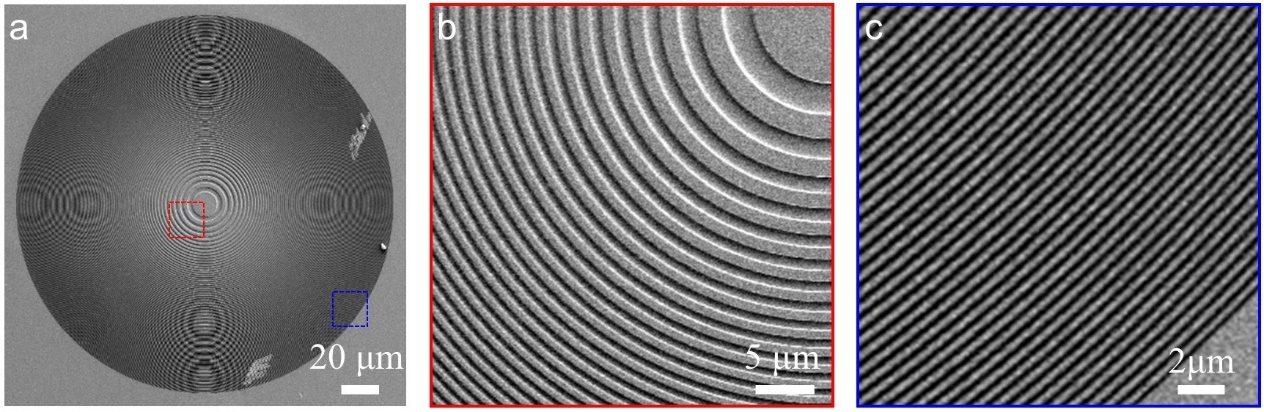
**

**Figure S10. SEM images of aplanatic Fresnel lens.** **a**, A top view of the lens. **b**, Zoomed view of the lens center. **c**, Zoomed view of the lens edge.

We first characterize the focusing property of Fresnel lenses. According to the diffraction principle, Fresnel lens will have a series of focuses with focal lengths of *f*, *f*/3, *f*/5 … both in transmission (real focus) and reflection (virtue focus), as schematically shown in **Fig. S11a**. **Figures S11b** and **S11c** shows the primary and secondary focal points respectively with the same exposure time. **Figures S11d** and **S11e** show the comparison of focus spots of real virtual primary focuses captured by CCD with the same exposure time as well. These multiple focuses undoubtedly indicate lower efficiencies than metalens that has only one focus. Moreover, by carefully analyzing the focus spots, we may find that achieved by Fresnel lens has sub-lobes feature, which is far from the ideal Gaussian profile as the metalens has, as the comparison results shown in **Figs. S11f** and **S11g.**


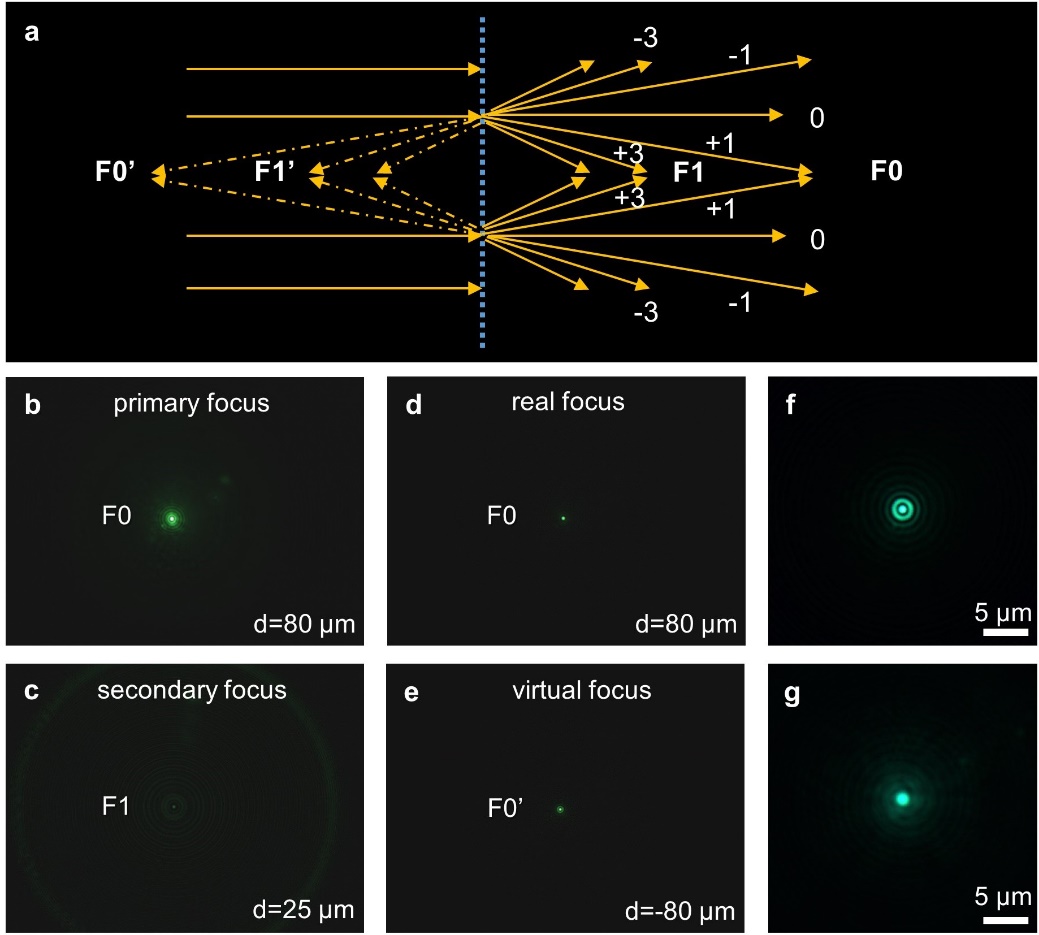


**Figure S11. Focus properties of Fresnel lens. a**, Schematic diagram of focusing for Fresnel lens. **b-c**, Primary focus and secondary focus. **d-e**, Real primary focus and virtual primary focus. **a**, Focus of Fresnel lens. **b**, Focus of metalens.

Next, we characterized and compared the resolution of four lenses (normal Fresnel lens, aplanatic Fresnel lens, normal metalens, aplanatic metalens). A positive 1951 United State Air Force (USAF) resolution test chart was directly mounted about *s*=2*f*=160 μm in front of the test lens, and was illuminated by a halogen light source with a *λ*=500 nm, *λ*=530 nm, and *λ*=560 nm filter (bandwidth=10 nm). According to the results shown in **Fig. S12,** the transverse resolutions of these former three lenses (normal Fresnel lens, aplanatic Fresnel lens, normal metalens) are 997 nm (element 1, group 9), while the aplanatic metalens exhibits a higher resolution of 775 nm (element 3, group 9).

**
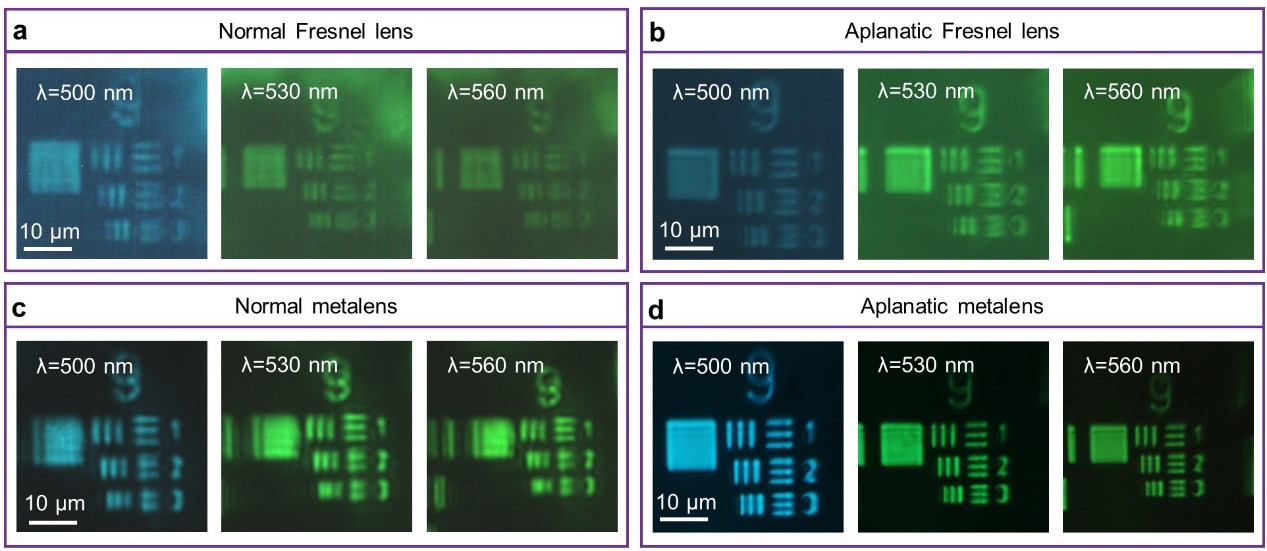
**

**Figure S12. Images of 1951 USAF resolution test chart with normal/aplanatic Fresnel lens and metalens. a-b,** Images of 1951 USAF resolution test chart with normal and aplanatic Fresnel lens. **c-d**, Images of 1951 USAF resolution test chart with normal and aplanatic metalens.

According to the same diffractive dispersion, Fresnel lens and metalens present similar broadband chromaticity. Their broadband imaging properties were also analyzed by a resolution test chart (a positive1951 USAF resolution test chart). **Figures S13a** and **S13b** show the images by aplanatic Fresnel lens and aplanatic metalens, respectively, where the wavelength ranges from 500 nm to 660 nm, and the magnifications of images are all about 1.8. To be noted, in experiments there were no polarizers used in Fresnel lens imaging because the lens is independent of the polarization. As a result, the image quality by Fresnel lens is much worse than that by metalens, especially in the signal-to-noise ratio (SNR). Taking the imaging under λ=530 nm as an example, we obtained the SNR of metalens is about 8.51 dB much higher than that of Fresnel lens (1.46 dB) (here SNR is defined by 10lg(*I*_signal_/*I*_noise_)), as depicted in **Figs. S13c** and **S13d**. It is rightly due to the fact that metalens imaging is polarization dependent, and the SNR is greatly improved by cross-polarization filtering through the metalens imaging. Significantly, this advantage of high SNR by metalens is further verified by the microscopic imaging of the frog egg cells. A same spectral tomographic imaging procedure was carried out with the aplanatic Fresnel lens in the same wavelength range. **Figure S14** shows the experimental tomography results with these two kinds of lenses, from which it is evident that PB phase designed metalens shows much better imaging quality in resolution, image sharpness and contrast. The advantage of metalens for such kind of spectral tomography is clearly demonstrated.

**
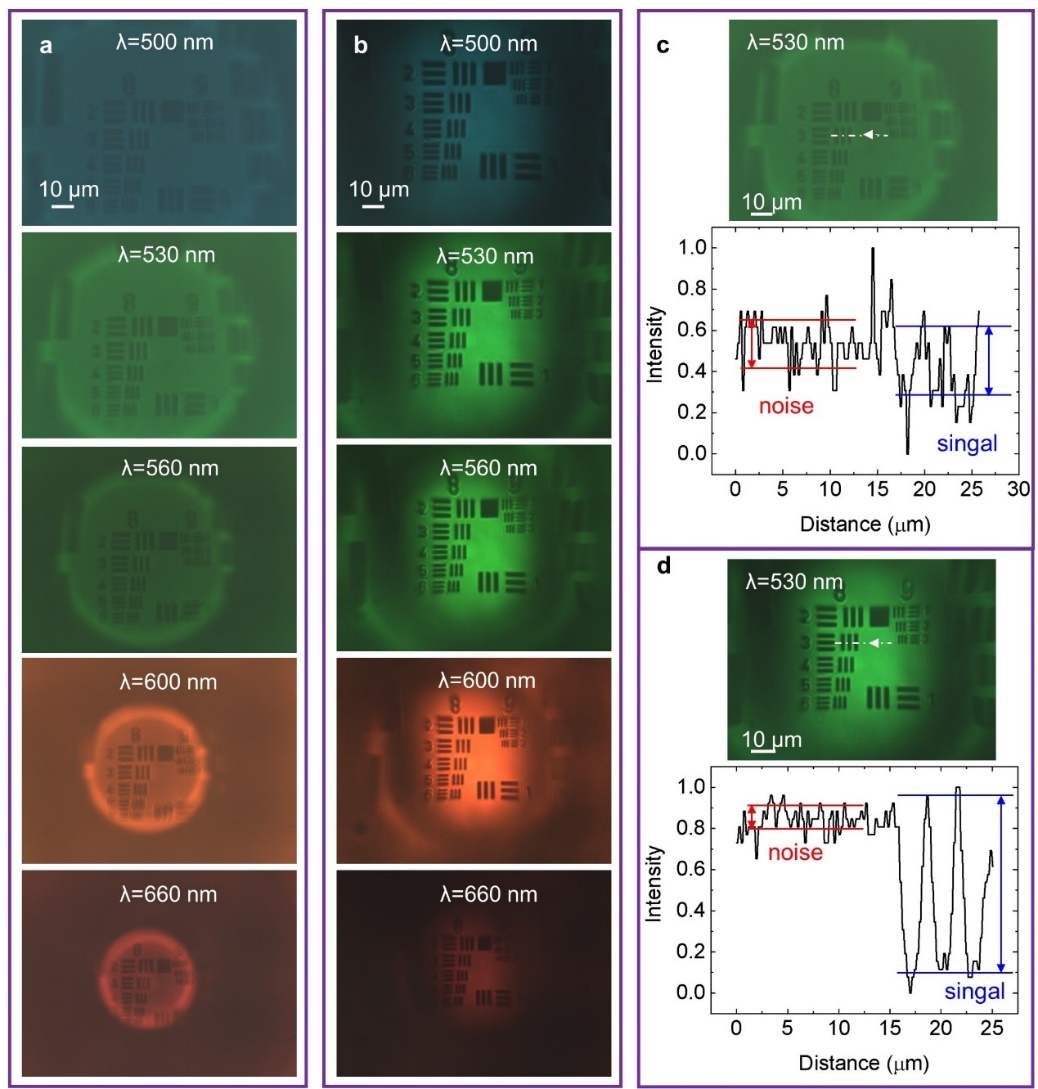
**

**Figure S13.** **a**, Images with aplanatic Fresnel lens. **b**, Images with aplanatic metalens. **c**, Signal-to-noise ratio of the aplanatic Fresnel lens, object is a positive 1951 USAF resolution test chart. **b**, Signal-to-noise ratio of the aplanatic metalens.

**
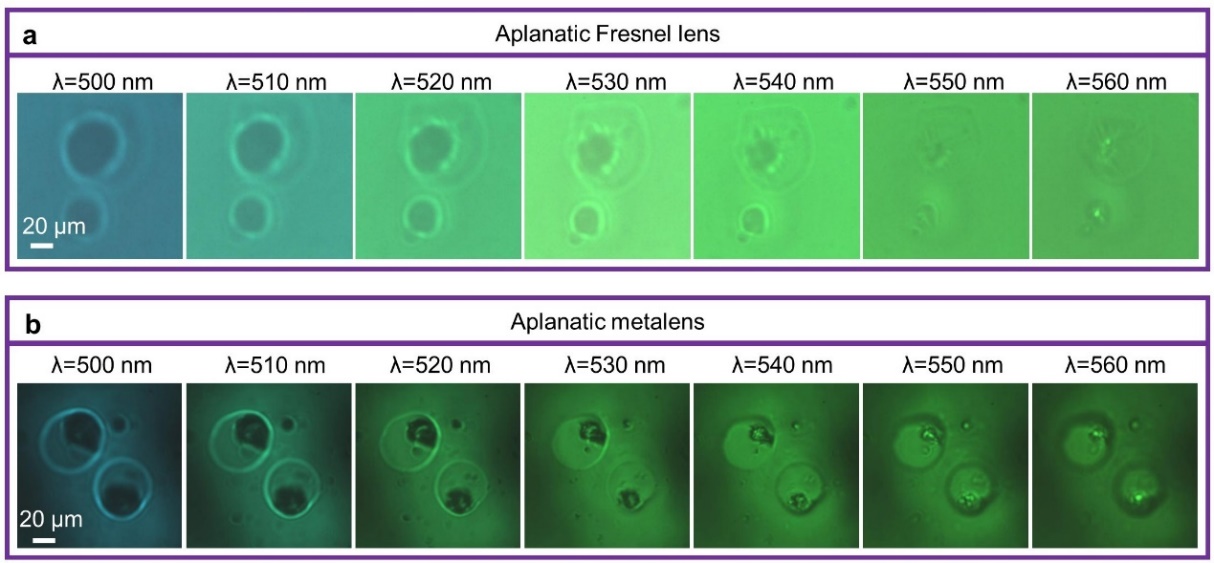
**

**Figure S14.** **Spectral tomographic images of Frog egg cells** with **a,** the aplanatic Fresnel lens, and **b,** the aplanatic metalens.

Moreover, we additionally carried out the full-wave simulations to analyze the working efficiencies of three types of Fresnel lenses by Lumerical-FDTD. **Figure S15a-c** show results of Fresnel lens with NA=0.78, including electric field distributions of focus plane and y-z cross section. The secondary focal points are observable in all three cases (including the binary, four-level, and eight-level Fresnel lens). The corresponding result of GaN metalens are also shown in **Fig. S15d**. Their transmittance, focusing efficiency (the ratio of the optical power in an area of diameter 3×FWHM to the total power in the focal plane) and total efficiency (transmittance×focusing efficiency) are calculated respectively, as summarized in **Fig. S16**. Apparently, the efficiency of metalens is higher even than that of all Fresnel lenses, though the eight-level Fresnel lens is closer. To be noted that high level Fresnel lenses are not easily fabricated, to get high efficient diffractive lens still remains a big challenge.


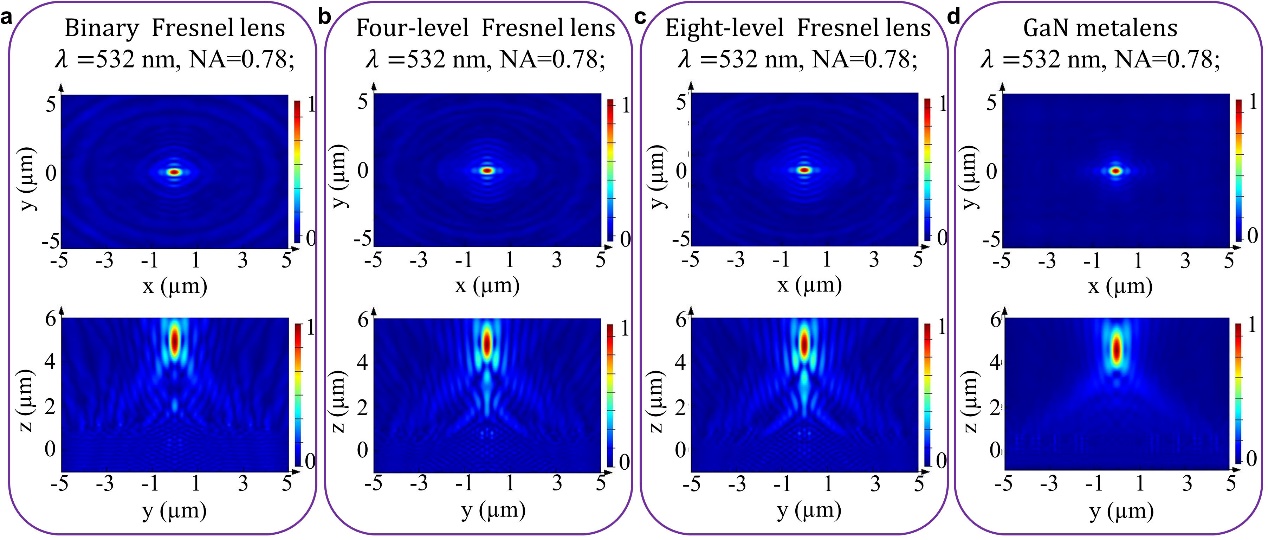


**Figure S15.** Electric field distributions of focus plane and y-z cross section of **a,** binary Fresnel lens. **b**, four-level Fresnel lens. **c**, eight-level Fresnel lens **d**, GaN metalens with NA=0.78.


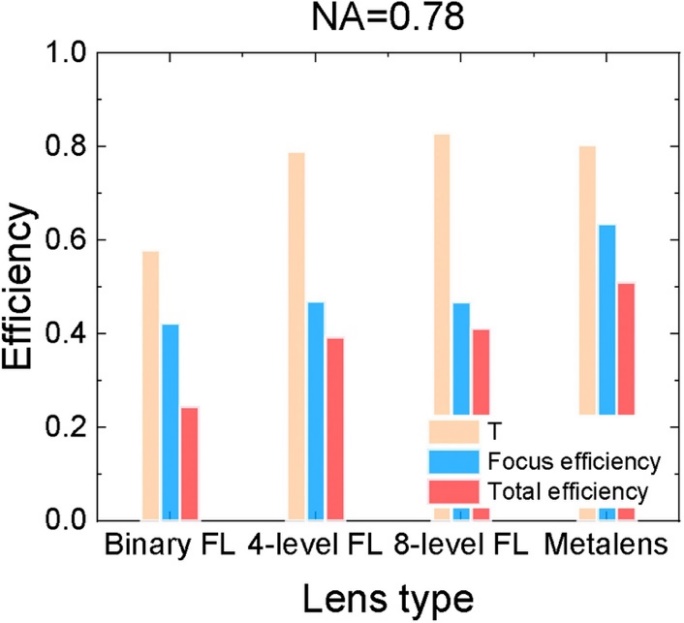


**Figure S16.** Efficiencies of binary, four-level, eight-level Fresnel lens, and GaN metalens with NA=0.78.
